# Observations on the Remarkable Efficacy of Carrots, under a New Mode of Application, in the Cure of Ulcers and Sores

**Published:** 1810-02

**Authors:** Richard Walker


### *Observations on the remarkable Efficacy of Carrots, under a new Mode of Application, in the Cure of Ulcers and Sores.* By Mr. RICHARD WALKER.

(From the Philosophical Magazine.)

THE carrot poultice is an application which has been long in use to correct the disposition and improve the discharge of the putrid or scorbutic ulcer.

The manner in which it is usually applied, is by grating or scraping the carrots fine, and laying them on raw.

I have lately had reason to believe, that the effects of it may be considerably increased, by varying the mode of application.

Several cases occurred in the Radcliffe infirmary, during the summer and beginning of the winter last year, of the true, malignant, scorbutic ulcer.

All the common methods of treatment were adopted, and of course the carrot poultice was not omitted.

The inefficacy of it however was too evident.

In consequence therefore of the ill success attending this practice, several of the cases terminating fatally, and as fresh instances were continually occurring, the following change was at length tried, in the use of this remedy.

The carrots being previously cleaned by scraping and washing, were split and boiled till quite tender, in a small quantity of water; the liquor was then strained, or poured off, and the carrots beaten in a mortar, to the consistence of an uniform soft moist pulp.

The ulcers were first washed clean with the liquor rather warm, in which the carrots had been boiled, sometimes fomented with it, and the carrot poultice being previously spread ready, that the sore might be as little exposed to the air as possible, applied cold.

This

This was repeated night and morning, and oftener when the quantity of discharge, or other circumstances, made it necessary; but this was seldom required, when the mode above mentioned was adopted sufficiently early; that is, before the sore had made much progress in its scorbutic state.

It scarcely need be observed, that this disposition was known to have taken place, when the ulcer, from being firm, florid, and discharging good pus, became spongy, pallid, and discharged a considerable quantity of a thin, bloody, or gleety kind of matter.

The superior effects of this treatment were apparent in a very short time; in a few days the sores (several of which, before, were spreading rapidly, threatening the lives of the patients) were obviously improved; and in short, without any interruption to their progress in amendment, they were all of them gradually restored to a healthy appearance; and the cure finished, either by a continuance of this, or the methods ordinarily used to sores in a healthful healing state.

In all the cases above alluded to, bark, opium, &c. were as usual administered.

Nothing, however, has been particularly stated with respect to the exhibition of such remedies, as the object of the present paper is merely to direct the attention of practitioners to the use of the carrot poultice, and to recommend, under the sanction of many successful cases, the mode of applying it above described.

*Oxford, November* 2, 1795.

---

*November* 30, 1803.

Since the above was written, a very considerable number of similar cases have at different times occurred, in which the efficacy of the carrot poultice, applied as above, has been abundantly confirmed; viz. very large sores, chiefly on the leg, extending in some instances from the knee to the

---

* The antiseptic power of the carrot poultice has been ascribed, I believe, to the carbonic acid gas which the sore is supposed to imbibe from it during its application; hence it might be inferred that the carrot was fittest for use in its raw state.

I am however rather inclined to impute the efficacy of the carrot to its mild anti-putrescent quality, depending chiefly on the pulpy saccharine matter it contains in common with other vegetables, but in greater abundance; meliorated and softened into the fittest consistence by boiling and pounding for application to the tender, irritable surface of ulcers, sores, inflamed skin, &c.

the ancle, originating from accidental injury, habitual ulcers likewise, surfaces of stumps, and other sores after operations, all having assumed the morbid disposition before mentioned. In every one of these cases, the carrot poultice has been the immediate and constant resource, and with the completest success.*

As the efficacy of carrot poultice in different sores, and the fittest mode of its application, have, occasionally, ever since its adoption, engaged my particular attention, viz. for a period of nearly ten years, I am now enabled to speak more confidently, and with greater precision on the subject, and shall therefore give a more particular detail of every circumstance relative to it; premising in addition to what I have before said respecting that morbid disposition of a sore which requires this remedy, that it is commonly preceded by a more than usual disposition in the sore to bleed on the slightest touch or motion, and very quickly after this appearance the diseased state alluded to follows.

The carrots are now cut in thin transverse slices (instead of being split) for boiling, and the poultice when ready, observing to have it as moist as it will admit of without the inconvenience of its running about, instead of being spread on the cloth, is applied wherever the situation of the part will allow, by laying it on in portions with the hand, filling up first the cavities lightly, and then laying a coating of it about the thickness or rather more than that of an ordinary poultice, over the whole surface, and considerably beyond the edges of the sore; pressing it close, smooth, and of an uniform thickness, quite to the edge of the poultice; otherwise it will become dry at the edge, and occasion some inconvenience in removing, by its adhesion.

The cloth or fine linen is then to be applied and pinned tight over it; and a short roller may be used in order to keep the poultice uniformly close, and prevent it from being displaced.†

The more recently the carrot poultice has been boiled and

* Large wounds and ulcers not unfrequently acquire an ill-conditioned state, notwithstanding the most skilful application of adhesive plaster, requiring a suspension of that mode of practice for a time.

† The method here described of applying the poultice was found convenient in very large sores with irregular surface; but in general it may be applied in the usual way spread on cloth, observing that the fresh poultice be ready to be applied immediately on the removal of the old one.

and prepared the fitter it is for use, therefore it is best when prepared immediately before using. But as the process of boiling the carrots sufficiently requires some time, enough may be made at once for two or three days consumption, but not longer, particularly in hot weather, when indeed it should be prepared daily; and when it is necessary to warm it for application, this is best effected by placing a bason containing it in a vessel of water over the fire.

It is particularly requisite that the carrot poultice be applied as moist as can be, in order that it may not become too dry by the next time of application.

As many of the cases in which it is applied are those in which the temperature of the body and the sore are considerably above, or hotter than the healthy temperature, particular care should be taken that in such cases the poultice be applied so as to produce in the patient a sensation of coolness; but in ordinary cases, a sensation of warmth.

Most cases require it to be applied twice in the day, viz. every morning and evening; and very few indeed require it oftener.

If the sore should require from its foulness to be washed at the time of dressing, it is best done by squeezing a sponge full of the liquor out of a bason containing it over the sore repeatedly (catching the foul liquor in a bowl) till cleansed; the outside should then be wiped dry to the edge; the sore itself, however, should on no account be touched with the sponge, but be cleansed with lint if necessary.*

The liquor may be that in which the carrots have been boiled, or in defect of that, milk and water or pure water, observing that its temperature be not hotter than the sore can bear with the most perfect ease to the patient. The washing may be omitted unless when the sore is very foul.†

The effect of the carrot poultice thus applied is to correct the fœtor or stench of ill-conditioned sores, and to reduce them to a perfectly healthy or good-conditioned state; moreover to thicken and diminish the discharge as well as correct it; hence it follows that it is particularly indicated in large sores with too thin or too copious a discharge.

When

* This precaution is particularly necessary in putrid cases, to avoid the danger of keeping up or renewing the contagion in the sore.

† It is essential that the sore be as little exposed to the air as possible; hence it is better not to be very solicitous in cleansing the sore, the repetition of the poultice effecting this sufficiently.

When the sore is found to be sufficiently restored by the use of the carrot poultice, it should be dressed by applying first a single stratum of loosely made lint, not of the close compact kind which is made by an instrument; then a pledget of any common simple cerate, spread fresh and rather thick on fine cloth, if the sore be very large, otherwise upon fine lint, sufficient to cover the edges of the sore completely, and over this a defensative plaster in the usual way of epulotic cerate on tow, with a compress and moderately tight roller. Dressing once a day is commonly sufficient, that is, every morning; but if the sore is large, or whilst the discharge is copious, it is better to dress it twice every day.

If the discharge is considerable, the stratum of dry lint upon the sore may be thicker, that is, in all instances just sufficient to absorb or retain the discharge.

It is not amiss, when the sore is become apparently fit for dressing, to apply one or two poultices more, having a single stratum of fine lint applied as above, immediately under the poultice, and then proceed as before mentioned.

The carrot poultice may be safely and efficaciously applied to sores in a healthful, healing state; but as sores then require pressure by bandage, and other management, known to every experienced surgeon, it is best to stop the use of it at this stage.

Since the effect of this carrot poultice is in a peculiar degree to diminish as well as thicken the discharge of a sore, it should never be used where an increased discharge is required, from mischief being likely to arise by pent-up matter; as when any part becomes swollen or inflamed for want of a free discharge at the sore, in that case a soft emollient poultice, and the practice usual in such cases, must be adopted.*

The

* Cases of this kind, in which alone its application is objectionable, cannot be confounded with the dry, foul, or scorbutic ulcer, in which the carrot poultice by correcting the disease promotes a healthy discharge, and separation of sloughs; nor with sloughs arising from various other causes, such as sometimes occur in the course of the cure of gun-shot wounds, burns, &c. in which it is equally efficacious. Unctuous applications to sores of large surface are apt to produce superficial sloughs, which increase, or spread, by continuing the use of such applications—this disposition not unfrequently occurs in extensive scalds. Where such sloughs, accompanied with intensely inflamed edges, are forming from this cause, it is truly astonishing to observe the effect of this specific application in arresting the progress of this disease, by the almost immediate vanishing of the inflammation, the quick separation of the sloughs, and the rapid progress of the sore to a healthy healing state.

The carrot poultice in this form is applicable to all other species of sore, viz. venereal, cancerous, scrophulous, &c. and will be found, with the aid of proper medicines, the best application for the purpose of keeping the sores in good condition, and healing such of them as are not in their nature incurable.

The carrot poultice as above, is a good application to excoriations of the skin in any part, or from any cause or disease where a thin disagreeable discharge occurs.

In the cases before mentioned, where the carrot poultice is improper from pent-up matter, if the surface of the sore has acquired the scorbutic taint, a thin stratum of the carrot poultice may be applied over that surface, and the emollient poultice* over it, until that disposition is corrected.

The carrot poultice, as may be naturally inferred from what has been said of it, may be applied with singular good effect to a variety of other diseases which produce a thin, hot, acrid humour on the part, viz. ophthalmia, herpes, &c.

In old habitual ulcers, the carrot poultice may be applied at any time when the sore is foul or ill-conditioned; and particularly when such a sore has a dry sordes on the surface, carrot poultice applied thin over that surface, and an emolient poultice over it of bread and milk, never fail to bring on quickly a discharge of good-conditioned pus.

It sometimes happens when a cure is tedious, as in sores of extensive surface, or of a languid or sluggish disposition, that from the mere changing of the application for another a short time, and then renewing the former, the sore will become invigorated and more disposed to heal than before; when this appears to be the case, the intervention of a few carrot poultices will effect it, I think, better than any other application, and hasten the healing of the sore very considerably.

Small obstinate sores in bad habits which resist the usual means, are commonly brought into a healing state by carrot poultice alone, but sometimes more readily when it is conjoined with the use of *hydrargyrus nitratus ruber*; and when such a sore is become clean and florid, the cure may be completed by dressing with a little of the down of lint loosely upon, or in the sore, and the carrot poultice over it.

There

* A poultice of bread and milk is, I believe, much fitter for this purpose than one of linseed flour.

There is no circumstance in the curative art more lightly, but more erroneously thought of than the healing of sores; this being supposed by many to depend upon the mere circumstance of taking off one plaster and putting on another; whereas too frequently even an apparently trifling sore (not arising from any constitutional cause and consequently requiring no internal medicine) will baffle for a long time the efforts of a skilful practitioner: and, indeed, I am well assured, that very commonly the patient is loaded with bark, &c. to the injury of his health; whilst the sore remains the same, or is becoming worse, till a mode of dressing appropriate to that particular case is hit upon.

Carrots may be procured fit for use all the year round, and though fittest when they have but just arrived at maturity, are nevertheless sufficiently efficacious at all seasons. Or they may be collected at the proper season, and preserved in sand, till the next return of them to a perfect state.

In defect of a pestle and mortar to pound the carrots, a wooden wash-hand bowl, with an appropriately-formed pestle of wood, having its base largely convex, in order to bruise the carrots more readily, may be used in their stead.

Of late years, bark and Port wine have been much more sparingly used in cases of scorbutic ulcers, &c. the carrot poultice, with an ordinary restorative diet, having been found to answer best.

In large sores that require a great quantity of the carrot poultice, the outer part of the poultice may be rather coarse, but that which applies to the sores should in all cases be a perfect pulp.

The only objectionable circumstance, that I know of, respecting the carrot poultice as an application, is its disposition to become dry, particularly when used in small quantities, as in small sores, or when the carrots are not in their most succulent, pulpy state: this circumstance, however, is completely obviated, by applying a stratum or portion of the prepared carrot upon the part affected, and laying a poultice over it of linseed flour, or bread and milk, as the nature of the case may seem to require.

I have been induced to offer these observations to the attention of the public, from a conviction of the utility that may ensue from the knowledge of the efficacy of the carrot poultice, thus prepared, being made general; which has hitherto, I have good reason to think, been chiefly confined to this vicinity; where this poultice is used as well in private practice as in the Infirmary, and with the most eminent advantage.

POSTSCRIPT.

At the time this mode was originally tried here, the usual, and I suppose I may say, the constant practice in surgery was, to apply the carrots raw as before mentioned; this manner of using them being directed in all books of surgery, and the practice of it confined chiefly to the purpose of removing the ill smell or fœtor of sores.

The circumstance that led to it was the extraordinary bad cases above related; which originated in a man who had a very large cancerous sore of the arm, which became so putrid and offensive, as to contaminate, as was supposed, the ward: several of the patients soon after, having sores, some even of a trifling description, which quickly assumed the putrid, scorbutic disposition above described, and several others in succession.

This affair became so serious, that it was thought advisable to have a consultation of the faculty, which accordingly took place.

The result of this was, all medical and chirurgical skill having been exhausted to no purpose, that all the wards should be fresh white-washed, and fumigated;——but still the evil continued with unabating fury.

At this juncture, having observed the effects of the carrot poultice used when raw, to exceed, in some degree, the rest of the various remedies employed, consisting, among others, of the fermenting poultice, so highly esteemed in cases of this nature; I proposed using a poultice made of the carrots boiled, hoping their efficacy might be increased thereby, attending particularly to the process and application myself; the result of which was, as before stated.

The good effects indeed of this treatment were so decided, that, although of sixteen cases which occurred in the course of the year 1794, ten terminated fatally, notwithstanding the most skilful application of the means then in use; there was not one, out of at least the same number of cases, equally dangerous, which presented themselves the year after, but what ended well under this new method.

Since that time this mode alone of applying the carrot poultice has been in use in the Radcliffe Infirmary, not only for the scorbutic or putrid kind of ulcer whenever it occurred, but for all untoward or foul sores of every description.

That the efficacy of the carrot poultice thus modified, is not generally known, even at this time, I can assert with some degree of confidence, having been repeatedly assured assured by a professional gentleman, that the carrot poultice, prepared in the old way, is still in general use, and without attributing any efficacy to it, beyond that which was originally allowed to that remedy. It has, however, lately found its way into some publications, but in a very vague and indeterminate manner.

*Oxford, Oct.* 1, 1806.

---

*** The above account of the efficacy of carrots, brings to the Editor's recollection, a similar instance of cure performed by turnips, as communicated by a friend. The following is the case alluded to: "A man about 50 years of age, and who had lived irregularly, had been for several years afflicted with ulcers on both legs. They at last extended from the knee to the ankle downwards, the discharge being greater, and the sores worse-conditioned along the shin-bone in front of the leg. When the writer of this article first saw the man in question, he was confined to bed, and had been unable to walk across the room for several weeks: he had been successively attended by all the medical gentlemen of the town in which he lived, and had undergone several courses of medicine with a view to purify the system, but without effect: his sores were dressed with the usual ointments. The application of turnip poultices was suggested to him by a country woman who came into the town on market-days. Her instructions were, that he should night and morning apply poultices of white turnips to the sores, previously bathing them with the liquor, squeezed out when the roots were boiled into pulp. The poultices were directed to be applied hot. The above directions were faithfully attended to by the patient under the inspection of the writer of this article: within the first twenty-four hours, the ulcershad assumed a different appearance, and in about a week from the first application of the turnips, the ulcers were so far healed that the man was able to walk out. In a few days afterwards, the sores entirely disappeared, and the skin soon resumed its usual appearance. During this period, no medicine was taken by the patient; the state of his bowels not even requiring a dose of salts."

